# Long Term Non-Invasive Ventilation in Children: Impact on Survival and Transition to Adult Care

**DOI:** 10.1371/journal.pone.0125839

**Published:** 2015-05-01

**Authors:** Michelle Chatwin, Hui-Leng Tan, Andrew Bush, Mark Rosenthal, Anita Kay Simonds

**Affiliations:** 1 Academic and Clinical Department of Sleep and Breathing, Royal Brompton & Harefield NHS Foundation Trust, Sydney Street, London, United Kingdom; 2 Department of Paediatrics, Royal Brompton & Harefield NHS Foundation Trust, Sydney Street, London, United Kingdom; 3 NIHR Respiratory Biomedical Research Unit, Royal Brompton & Harefield NHS Foundation Trust, Sydney Street, London, United Kingdom; University of Tübingen, GERMANY

## Abstract

**Background:**

The number of children receiving domiciliary ventilatory support has grown over the last few decades driven largely by the introduction and widening applications of non-invasive ventilation. Ventilatory support may be used with the intention of increasing survival, or to facilitate discharge home and/or to palliate symptoms. However, the outcome of this intervention and the number of children transitioning to adult care as a consequence of longer survival is not yet clear.

**Methods:**

In this retrospective cohort study, we analysed the outcome in children (<17 years) started on home NIV at Royal Brompton Hospital over an 18 year period 1993-2011. The aim was to establish for different diagnostic groups: survival rate, likelihood of early death depending on diagnosis or discontinuation of ventilation, and the proportion transitioning to adult care.

**Results:**

496 children were commenced on home non invasive ventilation; follow-up data were available in 449 (91%). Fifty six per cent (n=254) had neuromuscular disease. Ventilation was started at a median age (IQR) 10 (3-15) years. Thirteen percent (n=59) were less than 1 year old. Forty percent (n=181) have transitioned to adult care. Twenty four percent (n=109) of patients have died, and nine percent (n=42) were able to discontinue ventilatory support.

**Conclusion:**

Long term ventilation is associated with an increase in survival in a range of conditions leading to ventilatory failure in children, resulting in increasing numbers surviving to adulthood. This has significant implications for planning transition and adult care facilities.

## Introduction

Provision of long term ventilatory support in children is increasing in the UK [1]. Factors that may influence this are: a growing evidence base for efficacy, improvements in ventilator technology and interface design for children; and physician and public awareness. The majority of these children are managed at home [1,2]. Use of ventilatory support can lead to a decrease in admissions to the paediatric intensive care unit (PICU) [3] and facilitate discharge home after ventilatory decompensation [4]. While long term ventilatory support may be delivered invasively via tracheostomy (TIPPV) or non-invasively via mask ventilation (NIV), the greatest expansion has been in the use of NIV [1,5]. In patients with neuromuscular disease (NMD), respiratory insufficiency is the commonest cause of death, and before routine ventilatory assistance death frequently occurred in infancy or childhood [6,7,8]. The use of NIV to control respiratory failure has been shown to increase survival [4,9,10,11,12], and slow functional decline with no associated decrease in health related quality of life [13], but case series tend to be small and focus mainly on outcome in Duchenne muscular dystrophy (DMD) and spinal muscular atrophy (SMA). Furthermore, longer survival in some patients may lead to complications previously not encountered and/or progressive ventilator dependency [11,14]. Perhaps due to the success of NIV in patients with NMD, this treatment has been extended to other disease groups e.g. chronic lung disease, neurological and neurodevelopmental syndromes, and upper airway disorders associated with hypoventilation. Here too, outcomes are unclear.

As NIV supports ventilation in patients, respiratory failure may not be the primary cause of death. In DMD, cardiac failure is an increasingly common cause of death [15]. Complications of NIV include mid-facial hypoplasia from long term mask use in younger children in whom facial growth is not complete [16]. Pneumothorax has been reported in patients with DMD [17] and with an increases in life expectancy, deaths may occur from either a unrelated illness or new complications may occur e.g. nephrolithiasis [18] or bowel pseudo obstruction in adults with DMD [15].

With improvements in survival resulting in an increased prevalence of some conditions [11,19], transition of the ventilator dependent child to adult services and management of the evolution of the condition become important aspects of care for both paediatric and adult healthcare teams.

We hypothesised that the use of NIV would be associated with increasing numbers of children transitioning to adult services. Our centre operates a model of a continuum of care from birth to old age for all patients with heart and lung conditions and so transition occurs within the same hospital. In the largest series reported so far, we describe here our experience of children requiring long term ventilatory support with regard to the duration of ventilation and outcome after transition, with the goal of providing data to inform patients and their families about likely outcomes, and ensuring that health services plan care optimally for this new group of technology dependant adult patients. We also identify diagnosis groups where early death was seen, evaluate the reasons for discontinuation of ventilatory support and report long term complications related to long term ventilation in order to better understand long term care needs and complications.

For the purpose of this manuscript non-invasive ventilation and continuous positive airways pressure (CPAP) will be referred to as ventilatory support.

## Methods

Our centre has provided a National Health Service (NHS) home ventilation programme since 1987. Patients are usually referred to our centre via a consultant from their local hospital or in some cases by their general practitioner (GP). On referral, patients are reviewed by a specialist consultant adult or paediatric respiratory physician who assesses the individual, confirms indications for therapy and in conjunction with current guidelines [20] and the family, determines the ventilatory management plan.

### NIV and CPAP protocol

NIV is initiated in patients with a chronic elevation in daytime arterial carbon dioxide tension (CO_2_) (>6.5 kPa) i.e. established ventilatory failure. In this situation NIV is started without the requirement for nocturnal transcutaneous CO_2_ (TcCO_2_) and oximetry monitoring. The aim in this situation is to use NIV at night to correct diurnal CO_2_ level. If it was not possible to correct diurnal CO2 despite optimal nocturnal settings patients would be encouraged to use NIV for set periods in the day. In patients with daytime normocapnia but symptoms of sleep disordered breathing, or at high risk of this, a respiratory multi-channel sleep study is performed (Alice 4, Phillips Respironics, Bognor, UK; Somnologica for Embletta, Ottawa, Canada; Domino, Somnomedics, Randersaker, Germany) with TcCO2 monitoring (TINA and TCM4, Radiometer, West Sussex, UK.) The respiratory multi-channel sleep studies are scored manually in accordance with standard criteria [21,22]. Causes of abnormalities such as, adeno-tonsillar hypertrophy are investigated and addressed if indicated. In patients with an elevated nocturnal TcCO_2_ level (>6.5 kPa) for greater than 50% of the night, nocturnal NIV is the treatment of choice. Inspiratory positive airways pressure (IPAP) was increased until objectively there was an improvement in tidal volume or to the maximum the patient could tolerate. EPAP was set at a minimum of 4 cmH2O and increased where indicated in patients with upper airway abnormalities. Inspiratory time and respiratory rate per minute were set to be age appropriate and adjusted in patients with a high respiratory rate to be similar to spontaneous rate. Patients were first encouraged to increase time on NIV and then a sleep study was carried out on these settings. In the presence of an elevated CO2, further titration of settings occurred. In patients with a normal nocturnal TcCO2 level but evidence of an increased apnoea hypopnoea index (AHI) confirming obstructive sleep apnoea CPAP was started. Patients with OSA were managed with CPAP therapy when adenotonsillectomy was not an option (i.e. the patient was too high risk for surgery or it was felt they were too young) or in cases where the AHI was so severe it warranted treatment until the procedure was scheduled. In some cases CPAP therapy was started post adenotonsillectomy as a result of any residual OSA.

In SMA type 1 patients, treatment goals were set in collaboration with the family. NIV was provided to reduce dyspnoea and work of breathing, manage chest infections, prevent chest wall deformity and to facilitate discharge home. In a sub group of patients prolongation of life, in addition to symptom control, was the prime goal. The detailed ventilator management of this subset of patients is described in detail elsewhere [4].

A nasal mask or nasal pillows system was the interface of choice when initiating ventilatory support in the non-acute setting. In patients where optimal control could not be achieved with a nasal interface, the interface was switched to an oronasal mask. We used bilevel positive pressure ventilators or CPAP devices where indicated. Patients were initiated as either an inpatient or outpatient depending on severity of symptoms, patient preference and proximity to the hospital [23]. Prior to discharge a multidisciplinary plan is activated involving the community team. Home management of our service and maintenance program has previously been reported [24], all patients were managed with this service. Patients were followed up three months after discharge for a repeat sleep study on ventilatory support. If the sleep study results showed optimal ventilatory control, the patient was seen three months later in clinic. If the results of the sleep study were sub optimal, settings were adjusted and the patient returned for a repeat sleep study at the consulting physicians’ discretion. Stable long term patients usually received an annual sleep study and one clinic appointment per year. Patient and their families were provided with emergency contact details to enable them to arrange an urgent review if required.

We examined our hospital database to determine the number of patients aged 17 years or less in whom home non invasive ventilatiory support was initiated. Reference was made to: diagnosis, gender, age at initiation, and current age, to assess the outcome of patients that transitioned to adult services. We also investigated patient deaths where possible, and the reasons for cessation or withdrawal or progression of ventilatory support. We also investigated survival in a subset of Duchenne muscular dystrophy patients who were initiated on ventilatory support aged 18 or over. The aim of this was to investigate whether the age of which symptoms of ventilatory insufficiency ensues impacts on survival in this diagnosis group. This retrospective study of routinely collected clinical information was evaluated by the Research Office at the Royal Brompton and Harefield Hospital NHS Foundation Trust and did not require independent ethical approval. All data was obtained from clinical notes and no identifiable patient data is included, patient records were anonymised and de-identified prior to analysis (Hui-Leng Tan).

## Results

**Fig 1:**
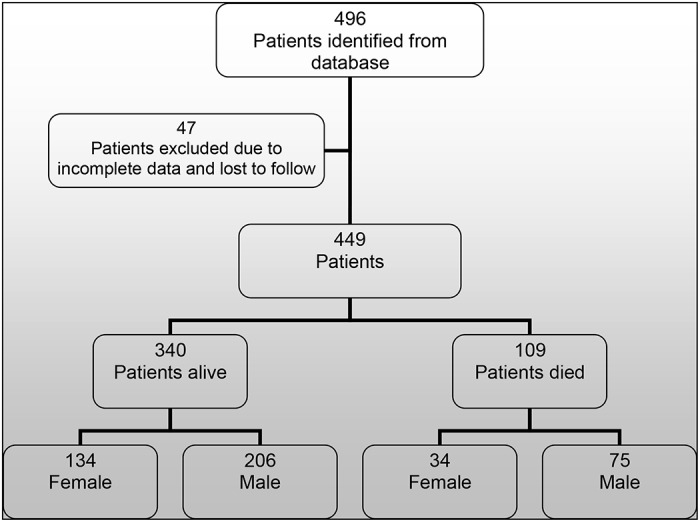
Consort Diagram. Fig 1 shows a CONSORT-style diagram for the patients who were identified, analysed and subsequent outcome.

Four hundred and ninety six patients were initiated on non invasive ventilatory support from January 1993 to December 2011. All records were cross referenced with the electronic patient record. Where there was no information or no documented follow up the records were excluded (n = 47). We therefore report the results of 449 patient records (Fig 1). For the cohort the mean age (SD) of initiation of patients receiving ventilatory support was 8.7 (6.1) years, median (IQR) age 10 (3–15) years. In SMA type 1 (n = 16) median (IQR) age at initiation of NIV was 6 (>1–12) months. The duration of ventilatory support for the cohort was a mean (range) of 74 (<1–221) months. Table 1 shows the total number of patients initiated on ventilatory support by age group.

**Table 1:** Patients with full data set initiated on ventilatory support by age.

Age (years)	Number
less than 1	59
1 to 3	75
4 to 5	32
6 to 9	57
10 to 17	226
Total	449

**Fig 2:**
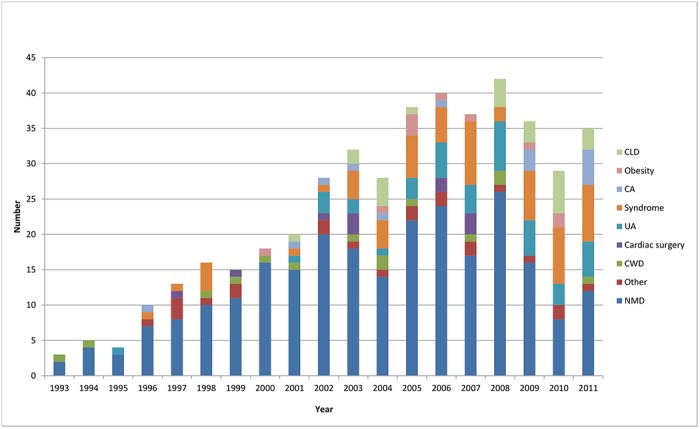
Patients initiated on ventilatory support by year and diagnosis. Fig 2 shows the number of patient’s initiated on ventilator support at our centre by year of initiation. It also shows the diagnosis group of the patient’s initiated each year. Note that over time the group diagnosis proportions change from predominantly neuromuscular disease (NMD) to include other such as chronic lung disease (CLD) and syndromes which include Trisomy 21. Central apnoea (CA), upper airway (UA) and chest wall disease (CWD).

**Fig 3:**
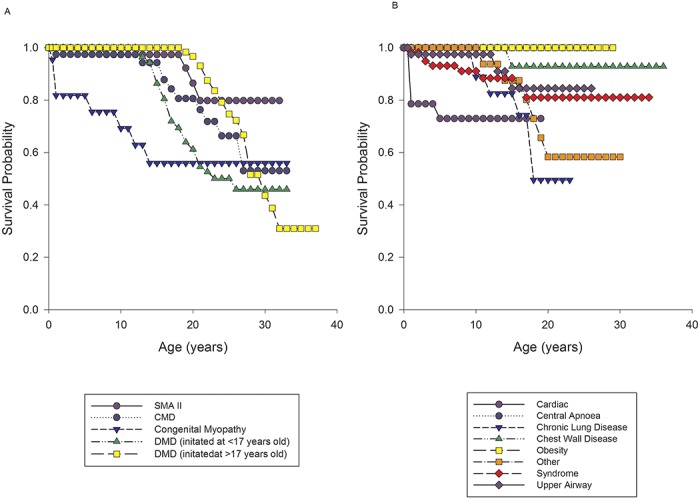
Survival probabilities for patients with neuromuscular disease who start non invasive ventilation. Fig 3A shows survival probabilities for; congenital muscular dystrophy (CMD); congenital myopathy; spinal muscular atrophy type II (SMA II); Duchenne muscular dystrophy (DMD) commenced on NIV < 17 years old; DMD commenced on NIV > 17 years old. Fig 3B shows survival probabilities for; air way abnormalities post cardiac surgery (cardiac); central apnoea; chronic lung disease; chest wall disease; obesity hypoventilation syndrome; other diseases not classified in any other group; syndrome and upper airway.

Patients were classified into groups (Fig 2 and Table 2). We found that there was a steady increase in patients receiving home ventilation until 2005, when referral of patients for long term ventilation stabilised (Fig 2). 392 patients received ventilatory support from a ventilator designed for home use and 57 patients used devices designed specifically for CPAP. No patient on CPAP was converted onto NIV. The majority of patients (76%, n = 340) were started on ventilatory support as inpatients, with 15% (n = 67) being started within PICU; we do not have an accurate record of the initiation site in 9% (n = 42). In the early years of the programme the predominant diagnosis of patients initiated with support was NMD. A likely explanation for this is due to the links with the Dubowitz neuromuscular centre at the Hammersmith Hospital. More recently there has been an increase in the number of patients with neurodevelopmental syndromes and CLD initiated on ventilatory support (Fig 2) reflecting a diverse referral population. 77% (n = 346) of patients only required 1 device, with the remaining 23% (n = 103) patients having 2 devices with internal and external batteries. 40% (n = 178) of patients had the highest priority level for replacing broken equipment as they had the greatest vulnerability. 10% (n = 43) had our second priority level, 38% (171) had the lowest priority level and 12% (57) were using CPAP which fell under a different provision for repairs. All children initiated on ventilation who were under a year old were provided with pulse oximetry monitoring (n = 59). The majority of patients were initiated on ventilatory support with a nasal interface (65%) with the remainder receiving an oronasal mask. The database current mean (SD) ventilator settings are: IPAP 18 (5.5) cmH_2_O, EPAP 6.0 (1.9) cmH_2_O, breaths per minute 18 (5.1) and CPAP setting are 8 (1.3) cmH_2_O. 28 patients required supplementary oxygen therapy with ventilator support. Specific diagnoses are shown in Table 3. Twenty patients all with neuromuscular disease; 8 with SMA type I, 3 with SMA type II, 7 with DMD, one myotybular myopathy and one with nemaline myopathy were provided with mechanical insufflation exsufflation devices. Survival and duration in patients with NMD: Survival curves for the neuromuscular group are shown in Fig 3. Age at initiation and length of time on ventilator support for patients alive and dead are shown in Table 4.

**Table 2:** Patients under the age of 17 who were commenced and ceased NIV and CPAP by group diagnosis.

Diagnosis Group	Total patients% (n)	Total patients onNIV% (n)	Total patientson CPAP% (n)	Total patients alive and discontinued NIV% (n)	Total patients alive and discontinued CPAP% (n)	Total patients who died using NIV% (n)	Total patientswho died using CPAP% (n)
Chest wall disease	4 (16)	100 (16)	0 (0)	13 (2)	0 (0)	6 (1)	0 (0)
Obesity	3 (12)	75 (9)	25 (3)	8 (1)	16 (2)	0 (0)	0 (0)
Chronic lung disease	5 (24)	87 (21)	13 (3)	25 (6)	0 (0)	21 (5)	4 (1)
Central sleep apnoea	3 (12)	100 (12)	0 (0)	0 (0)	0 (0)	0 (0)	0 (0)
Cardiac surgery	2 (11)	36 (4)	64 (7)	0 (0)	36 (4)	0 (0)	36 (4)
Congenital syndrome	14 (61)	56 (34)	44 (27)	7 (4)	5 (3)	10 (6)	5 (3)
Upper airway abnormality	9 (39)	62 (24)	38 (15)	10 (4)	15 (6)	5 (2)	2.5 (1)
Other	4 (20)	95 (19)	5 (1)	25 (5)	0 (0)	50 (10)	0 (0)
Neuromuscular disease	56 (254)	96.6 (253)	0.4 (1)	2 (4)	0 (1)	30 (76)	0 (0)

Non invasive ventilation (NIV), continuions positive airways pressure (CPAP). Cardiac surgery (post cardiac surgery requirement for ventilatory support); congenital syndrome includes Trisomy 21)The diagnostic breakdown of our NMD population included DMD 34% (n = 88); SMA 26% (n = 69); congenital muscular dystrophy (CMD) 15% (n = 38); congenital myopathy (CM) 11% (n = 11); congenital myasthenia syndrome (CMS) 4% (n = 11); hereditary sensory motor neuropathy (HSMN) 2% (n = 4); myotonic dystrophy 2% (n = 4); facioscapulohumeral muscular dystrophy (FSHD) 1% (n = 1)); autoimmune myasthenia gravis 1%, (n = 1) and other NMD 4% (n = 9).

**Table 3:** Number of patients who required oxygen therapy by disease group and individual diagnosis.

Diagnosis Group	Number requiring supplementary oxygen therapy	Sub diagnosis	Number requiring supplementary oxygen therapy
Chest wall disease	2		
Obesity	3	with pulmonary hypertension	1
		Prada-Willi	2
Chronic lung disease	8	Cystic fibrosis	3
		CLD of ex prematurity	2
		Lung fibrosis	1
		Obliterate bronchiolitis	1
		with pulmonary hypertension	1
Cardiac surgery	4		
Congenital syndrome	3	Retts syndrome	1
		Trisomy 21	2
Other	5	Fredrick’s ataxia	1
		Hypoxic brain injury	1
		Cerebral palsy	1
		Infantile neuroaxonal dystrophy	2
Neuromuscular disease	3	Myotonic dystrophy	1
		Spinal muscular atrophy type I	1
		Central core myopathy	1

**Table 4:** Age and length of time of ventilatory support by neuromuscular diagnoses.

Diagnosis (number)	Median age at initiation (years) (IQR)	Median length of time (months) on NIV (IQR)	median age of those alive (years) (IQR)	Median length of time (months) on NIV (IQR) of individuals who have died
SMA Type I (16)	0.5 (0–1)	33 (6–58)	4 (4–8)	6 (2–34)
SMA Type II (49)	7 (4–13)	68 (42–108)	14 (10–22)	44 (43–44)
CMD (39)	12 (9–14)	107 (50–147)	22 (13–25)	60 (10–95)
DMD initiated < 17 years old (88)	15 (14–16)	56 (31–90)	21 (19–25)	32 (13–63)
DMD initiated > 17 years old (63)	21 (19–23)	86 (48–113)	28 (24–31)	42 (15–70)
Congenital myopathy (22)	1.5 (0–8)	71 (49–117)	15 (7–19)	17 (1–54)
NMD group < 17 years old (254)	12 (5–15)	63 (36–107)	19 (12–23)	35 (6–63)

Spinal muscular atrophy (SMA), congenital muscular dystrophy (CMD), Duchenne muscular dystrophy (DMD), neuromuscular disease (NMD), interquartile range (IQR).

### Daytime ventilator use

82 of the 449 home ventilator patients (18%) used their ventilator during the day. Nearly all were neuromuscular patients who had progressed from nocturnal use, or were small infants requiring support during sleep or for ventilatory insufficiency during the day (e.g. SMA type 1 infants). Four NMD patients transitioned from NIV to TIPPV, in all but one case because of poor swallowing function which could not be managed with a combination of NIV, mechanical insufflation/exsufflation and percutaneous feeding. The diagnosis of these four patients were: Nemaline myopathy, myotubular myopathy, Duchenne muscular dystrophy and central core myopathy. One patient with cerebral palsy who had upper airway swallowing dysfunction progressed also to tracheostomy ventilation.

### Transition to adult care

Forty per cent (181 patients) have transitioned to adult services.

### Deaths

109 patients in the cohort have died; deaths in each diagnostic group for NIV and CPAP are shown in Table 2. Median (IQR) age of initiation of ventilator support of the cohort that died was 13 (4–15) years old and age at death was 15 (7–18) years old. Patients were on ventilatory support for 25 (6–55) months. Thirty two per cent (n = 35) of the patients who died had transitioned to adult services. Ten patients died at our centre, diagnoses were; cystic fibrosis (n = 3); NMD (n = 5), and two died of congenital cardiac disease. Causes of death and location are shown in Table 5. Only six per cent of the patients who died were receiving CPAP.

**Table 5:** Number of deaths by location along with cause of death.

	Number	Cause of death	Number
Deaths at our centre	10	End stage respiratory failure	3
		Cardiac failure	2
		Heart failure	2
		Sudden death in sleep	1
		Palliative extubation	1
		Hypoxic brain injury post aspiration of foreign body	1
Deaths at home	14	Expected death from respiratory failure with palliative care	4
		Sudden unexplained death	9
		Power failure	1
Deaths in hospice	1	Expected death from respiratory failure with palliative care	1
Deaths in hospitals elsewhere	30	Rapidly deteriorating severe NMD	5
		Bowel necrosis	2
		Tumour	1
		Respiratory failure	9
		Heart failure	4
		Anaesthetic complication	1
		Poor nutrition	1
		Unknown	7

### Long term complications

Complications related to long term survival/ NIV were rare before the age of 17 years. However in those > 17 years, three DMD patients developed a pneumothorax whilst on NIV, which was managed with simple tube drainage while continuing NIV, two developed acute gastric/bowel necrosis (1 DMD and 1 CMD), two (1 DMD and 1 SMA) experienced acute lactic acidosis at time of chest infections, two developed renal/bladder calculi requiring surgical removal and one DMD patient required ablation of an aberrant atrial conduction pathway for supraventricular tachycardia.

### Discontinuation of long term ventilation

Nine percent (n = 42) of patients have stopped using ventilatory support (Table 2). Some patients with NMD discontinued ventilatory support as a result of being unable to tolerate any airflow from the ventilator but were able to wear a mask (ventilator intolerance) (n = 5) (all had a diagnosis of SMA type II) and one patient with an atypical nemaline myopathy required ventilatory support from the age of 4 months to 5 years old, but was then able to maintain normal blood gas tensions during sleep and by day. Four patients did not tolerate any mask in contact with their face despite intensive psychological and play specialist support—each of these had severe learning difficulties with upper airway compromise. These patients were managed symptomatically and none required tracheostomy ventilation.

## Discussion

This is the largest cohort of children receiving non invasive long term ventilation ever to be described at a single centre and provides survival and discontinuation of ventilatory support information above and beyond that reported in single point survey studies. It also shows that around a third of DMD patients will survive into their 30s and early 40s; moreover, children with other neuromuscular conditions such congenital myopathies and muscular dystrophies have a significant probability of reaching adulthood. This increased survival can be associated with a range of complications not previously seen in childhood, and has important implications for healthcare planning.

Before the advent of ventilatory support infants with SMA type 1 died before the age of 2 years [25]. While gains in survival may not be the primary aim in this group, it has become clear that there is a spectrum of children with SMA type 1 and treatment with ventilator support will increase survival [26]. In those presenting at only a few months of age, symptom management may be most appropriate, whereas in those starting on NIV after 9 months longer term survival beyond 2 years to school age is more likely. Age at initiation of NIV is a prognostic factor as in DMD those requiring NIV before the age of 17 years had a worse outcome than those starting after the age of 17 years. Improvements in DMD care including scoliosis surgery, steroid therapy, physiotherapy and treatment of cardiomyopathy may delay the need for NIV.

Outcome has improved in other congenital muscular dystrophies and myopathies, though the heterogeneous nature of these disorders makes historical comparisons of survival difficult. Foley et al., [27] have shown that genotype-phenotype relationships in Collagen 6 related myopathies (which include Ullrich CMD, Bethlem myopathy and an intermediate variant) can predict respiratory outcome in that Ullrich patients require NIV at a mean (SD) of 11.3 (4.0) years, while in the Bethlem myopathy group ventilatory decompensation does not occur until adulthood. Our patient cohort contributed to the database of this paper [27]. In congenital myasthenia those with CHAT or RAPSYN mutations tend to develop apnoeas but only require NIV during acute exacerbations whereas in other mutations e.g. patients with COLQ may develop nocturnal hypoventilation and require long term home NIV [28]. Patients with genetic diagnostic confirmation in our cohort had COLQ (n = 4), CHAT (n = 1), Rapsyn (n = 2), CHERNE (n = 1) and DOK7 (n-1) mutations. In the future a clearer understanding of these genotype-phenotype relationships may aid prediction of the development of respiratory insufficiency and facilitate care.

Our patients with DMD died of cardiac or respiratory causes and this is similar to the findings of the Netherlands group [29] despite being managed by recommended guidelines [30]. We also experienced new issues in our older DMD patients which included: bowel necrosis in three patients, symptomatic nephrolithiasis [18] in three patients and pneumothorax in four [17]. These problems should be anticipated so that early recognition and prompt therapy minimise further complications, and as new issues with pain related to large joints and lumbar discomfort emerge, palliative care should become a crucial part of management. It should be borne in mind that symptom palliation may be required for years and that a palliative approach is compatible with active intervention e.g. resuscitation or PICU admission during acute severe chest infections. We received five reports of sudden death in sleep in patients with NMD without any reported illness or explanation in the period preceding the event, nor any evidence of ventilator malfunction. We believe these deaths may be due to acute sputum retention, autonomic dysfunction or sudden cardiac arrhythmia, but cannot prove this. Further investigation of this problem is required.

It is very difficult to provide the exact prevalence data on children receiving all forms of ventilatory support. Often reports include patients on tracheostomy ventilation. In accordance with others [1,12,29] we found that the number of children receiving home mechanical ventilation is increasing. In a UK based survey there was an increase from 141 children in 1998 to 933 in 2008 receiving long term ventilation across 30 regional centres [1]. McDougall and co-workers [12] in Canada initiated 144 patients on ventilator support between 1995 and 2009. They reported a plateau in the number of patients initiated on ventilation from 2002 (n = 14) with a peak in 2008 at 19 decreasing to 15 patients in 2009, although numbers are small, so trends difficult to assess. Paulides and co-workers [29] reported an increase from 8 patients initiated between 1979–1988 to 122 initiated between 1999–2008. They also reported a threefold increase in initiation in the younger group (age 0–5). Goodwin et al., [31] also reported a plateau in the number of patients to commence long term ventilation in their region of the UK with around 14 patients started on ventiltory support a year from 2005–2009. We too saw a steady increase in the number of patients initiated on ventilator support with increases in the 0–5 year old population. One explanation for the plateau in patients initiated on ventilator support is that in our area (London) there are now more specialist centres initiating children on ventilator support. A second explanation is numbers of patients with NMD constituted a decreasing proportion of the total number of patients initiated on NIV. A likely explanation for this is that NIV was initially offered to patients with NMD over other disease groups as this was the key area of research at the time. As more patients were initiated with ventilator support the prevalence of patients on ventilator support with a range of other disorders increased whilst the incidence of NMD remains the same. So, ventilation in other diagnostic groups has led to an equilibrium.

Unlike neuromuscular patients it is important to note that other diagnostic groups may be more likely to discontinue ventilator support. In our cohort reasons for discontinuation of ventilation included: resolution of tracheobronchomalacia and following cardiac surgery, which has also previously been reported [31]. Patients with CLD also ceased ventilatory support due to improvement in gas exchange. These improvements were due to lung transplantation in one patient with cystic fibrosis, and spontaneous improvement in four children with CLD of prematurity. Three obese patients achieved significant weight loss which resulted in resolution of symptoms and sleep disordered breathing, seven patients with trisomy 21 ceased support due to improvement in upper airway function. Five patients with upper airway obstruction improved following adenotonsillectomy, one patient improved spontaneously with age. In these diagnosis groups it is important to reassess the ventilator requirement. We have shown that there can be improvements in upper airway abnormalities that occur with growth/age Also where appropriate adenotonsillectomy may correct the sleep disordered breathing.

We acknowledge there are limitations to our data; we had a referral bias for NMD in the early years. Also some patients have been lost to follow up, we only included patients who were initiated on NIV and report the few cases that subsequently required tracheostomy ventilation to illustrate that this may be an appropriate treatment option in certain patients. We cannot provide accurate data on acute hospital admissions, nor give exact numbers of pulse oximeters provided in our high risk older children. We do not know the causes of death in all patients. Our data base also records a limited amount of information on interfaces as these were changed frequently to aid comfort and optimise ventilator efficacy as the child grew. Furthermore different interfaces became available on the market over time. We also are unable to report the exact number of patients who had midfacial hypoplasia, skin irritation and nasal dryness. However, in patients where patients were showing signs of facial abnormalities alternative masks were offered and rotated rather than customising masks as in other centres [16]. When skin irritation was a problem masks were lined with towelling or another barrier dressing and humidification was provided in the presence of dryness.

Like others [12,31] we report the number of patients transitioning to adult services is increasing. Forty percent of our cohort have transitioned to adult care compared to 17% [31] and 26% [12] in other studies. We also have a unique model of care, in that we look after patients from birth to old age. Transition is therefore from an inpatient or out patient setting with the medical team within the same hospital. This allows immediate communication between the adult and paediatric services along with streamlining services. Smaller centres may not be able to replicate this care pathway. However, our experience adds to the data published in this area and will help to provide further information on care approaches in this ever growing population.

Paulides and co-workers [29] reported that over the decades the number of patients who received invasive ventilation declined to 39%, as more children were started de novo on NIV. However, the proportion receiving invasive ventilation (51%) is higher than those at our centre (1%). It has been documented previously that the UK has fewer patients receiving invasive ventilation compared to other European countries [32]. The intensive use of mechanical insufflation-exsufflation combined with NIV and percutaneous feeding has also allowed us to continue with a non-invasive ventilatory approach in some patients who might have received tracheostomy ventilation previously [33], In addition as a centre experienced in home non-invasive ventilation, we may be preferentially referred these patients who are suitable for NIV, as opposed to receiving referrals of children requiring invasive ventilation.

Racca and co-workers [2] reported the outcome of 378 ventilator dependent children from 30 Italian centres of which 48% had TIPPV. They also had a heterogeneous population with a large proportion of patients with NMD (50%) and also successfully initiated ventilation in patients with cerebral palsy, upper airway disorders, CCHS, CLD and CWD. Unlike the Italian survey we did not use volume cycled ventilation in our children. This highlights difference in practice within specialist centres of Europe, but highlights that successful initiation of children is down to a high level of expertise and not the type of ventilator used.

Like others [2,19,29] we believe that the best outcome for patients receiving home mechanical ventilation comes from specialist centres which have the collective experience to identify and solve the wide variety of problems these patients may experience, together with the resources to do this. It is important to note that as the number using home mechanical ventilation has grown over decades [1,2,12,19,29] with increased survival [4,9,10,11] guidelines have been published to optimise patient care [20]. It is hoped that by outlining proactive management plans, patients who require ventilatory support can be treated prior to an acute admission to the PICU, thereby avoiding the complications and cost of a PICU admission. The lack of transition facilities for chronic neuromuscular disorders has been highlighted [34], and this report supports the extension of these.

In conclusion, home ventilation has become more feasible in children with a range of disorders leading to ventilatory failure. An understanding of the change in outcome that occurs as a consequence should help the multidisciplinary team and family/patient manage expectations, facilitate anticipatory care, and plan resources for this needy group when they transition to adult care.
